# Measurement and modeling of rizatriptan in supercritical CO_2_ for pharmaceutical processing

**DOI:** 10.1038/s41598-025-26435-w

**Published:** 2025-11-26

**Authors:** Sami Bawazeer

**Affiliations:** Department of Pharmaceutical Sciences, College of Pharmacy, Umm Al-Qura University, Makkah, Kingdom of Saudi Arabia

**Keywords:** Rizatriptan, Supercritical carbon dioxide, Solubility, Thermodynamic modeling, Chemistry, Engineering

## Abstract

This study examines the solubility of rizatriptan, a migraine medication, in supercritical carbon dioxide. The goal is to promote environmentally friendly drug manufacturing methods, such as antisolvent precipitation via supercritical fluids. Solubility levels were evaluated at temperatures ranging from 308.2 K to 338.2 K and pressures ranging from 12 to 30 MPa. These evaluations produced mole fraction values ranging from 0.24 × 10^−4^ to 4.19 × 10^−4^ and dissolution concentrations ranging from 0.008 g/L to 0.302 g/L. An estimated crossover pressure of approximately 15 MPa was identified, resulting from the interaction between the compound’s vapor pressure and the fluid’s density. Seven density-based models such as Chrastil, Bartle, Kumar–Johnston (K–J), and Mendez–Santiago–Teja (MST), Bian et al. and Sodeifian et al. (Ⅰ and Ⅱ) models were implemented. The Sodeifian et al. model (Ⅱ) demonstrated the strongest alignment (AARD = 7.78%, R^2^ = 0.990). Key extracted thermodynamic metrics include the overall enthalpy (38.06 kJ/mol), the enthalpy associated with vaporization (50.55 kJ/mol), and the enthalpy linked to solvation (− 12.49 kJ/mol). Additionally, three thermodynamic equations Peng–Robinson, Soave–Redlich–Kwong, and regular solution were examined. The regular solution model produced the best results (AARD = 6.89%, R^2^ = 0.991). These results provide reliable data and simulation models that can improve supercritical carbon dioxide methodologies, and advance eco-friendly drug production strategies.

**Supplementary Information:**

The online version contains supplementary material available at 10.1038/s41598-025-26435-w.

## Introduction

 Rizatriptan benzoate is used to treat migraines with or without aura. It provides rapid pain relief within two hours. However, the bioavailability of conventional oral tablets is limited to 40–45% due to first-pass metabolism and slower dissolution. This subject is exacerbated by the nausea and delayed gastric emptying that occur during migraine attacks. To address these challenges and enhance bioavailability, novel formulations have been developed, including oral disintegrating strips, mouth-disintegrating tablets, and fast-dissolving films. These advanced delivery systems disintegrate within seconds to minutes, achieving 95–100% drug release within 5–10 min in simulated salivary fluid (pH 6.8) or acidic media (pH 1.2) a significant improvement over conventional tablets, which release only 80–90% of the drug within 30 min. Their large surface area, enabled by thin films or porous structures, and amorphous drug dispersion facilitate rapid dissolution and absorption^1–5^.

These formulations improve bioavailability, achieving an area under the curve that is up to 67% higher than that of tablets and reducing time to reach maximum concentration to as little as 1 h. This ensures quicker migraine relief. They also enhance patient compliance, particularly among pediatric and elderly populations, by eliminating the need for water and enabling discreet administration. These innovations create highly effective, novel delivery systems that optimize the therapeutic efficacy of rizatriptan for managing acute migraines.

Particle engineering is pivotal for optimizing the physicochemical properties of active pharmaceutical ingredients (APIs), such as rizatriptan, to enhance solubility and bioavailability. Traditional methods such as milling or solvent-based recrystallization often yield broad particle size distributions, risk polymorphic transformations that may compromise drug stability, and introduce residual solvent contamination, posing regulatory challenges. Supercritical fluid (SCF) technologies, particularly those utilizing supercritical carbon dioxide (SC-CO_2_), offer a sustainable alternative due to CO_2_’s non-toxic, non-flammable nature, low cost, and mild critical conditions^6–9^. SC-CO_2_’s tunable solvent properties, modulated by pressure and temperature, enable precise control over crystallization, particle size reduction, and morphology, making it ideal for thermally labile compounds like rizatriptan. Applications include determining the optimal method based on the solubility of drugs in carbon dioxide, as in RESS and GAS^10–15^.

Determining solubility in SCFs, particularly SC-CO_2_, depends on two key experimental aspects: the method used to saturate the solute and the technique used to measure concentration. The saturation process is typically carried out using either the static or dynamic method. In the static approach, the solute and SCF are placed in a closed equilibrium cell under controlled temperature and pressure until equilibrium is reached. After equilibration, the supercritical phase is sampled and analyzed to determine solubility. The static method is simple, reliable, and well-suited for systems with low solubility or slow mass transfer. Conversely, the dynamic method involves flowing SC-CO_2_ continuously through a fixed bed of solute under constant conditions. The solute-laden fluid leaving the cell is analyzed until steady-state concentrations are achieved, representing equilibrium solubility. Although the dynamic approach enables faster equilibration and is preferable for volatile compounds, it requires more complex instrumentation and precise flow regulation. The second aspect concerns the analytical techniques used to quantify solute concentration in the saturated phase. Common methods include gravimetric, chromatographic, and spectrometric analyses^16–18^. The gravimetric method is the most widely used due to its simplicity and accuracy. It involves depressurizing and weighing the recovered solute. As Bartle et al.^19^ noted, this technique has historically been predominant. Chromatographic methods, such as gas chromatography (GC) and high-performance liquid chromatography (HPLC), offer greater sensitivity and selectivity, particularly for multicomponent systems. Recently, spectrophotometric approaches have been used to determine solubility in SC-CO_2_, as demonstrated by Sodeifian et al.^20–27^.

Determining solubility in supercritical fluids is often costly and time-consuming. This has prompted the development of theoretical models, such as empirical, equation of state (EoS), and expanded liquid models. Empirical models are popular due to their simplicity and reasonable accuracy within experimental conditions; however, they lack predictive capability beyond the measured data. In contrast, EoS based models, including cubic (SRK and PR) and non-cubic (SAFT) equations, use fugacity coefficients to provide better extrapolation capabilities^28–30^. Some researchers treat SCFs as condensed gases, while others consider them expanded liquids due to their high density. This leads to models such as UNIQUAC and the modified Wilson model, which are based on activity coefficients^31,32^. The effectiveness of each approach depends on the conditions of the system (temperature and pressure) and the properties of the compound. Overall, empirical models offer practical correlations within data limits, while EoS-based models are superior for broader predictive applications.

This study thoroughly examines the solubility of rizatriptan in SC-CO_2_ for pharmaceutical processing by supercritical fluid technology. A series of experimental solubility measurements were conducted at a range of temperatures, from 308.2 to 338.2 K, and pressures ranging from 12 to 30 megapascals. Seven density-based models ( Chrastil, Bartle, K–J, Méndez–Santiago–Teja, Bian et al., and Sodeifian et al. (Ⅰ and Ⅱ)) and three equations of state (Peng–Robinson, Soave–Redlich–Kwong, and regular solution) were employed to analyze the resulting data. Additionally, the enthalpies of the total, vaporization, and solvation processes were derived to elucidate phase behavior.

## Materials and methods

### Materials

The all information were reported in Table 1.

**Table 1:**
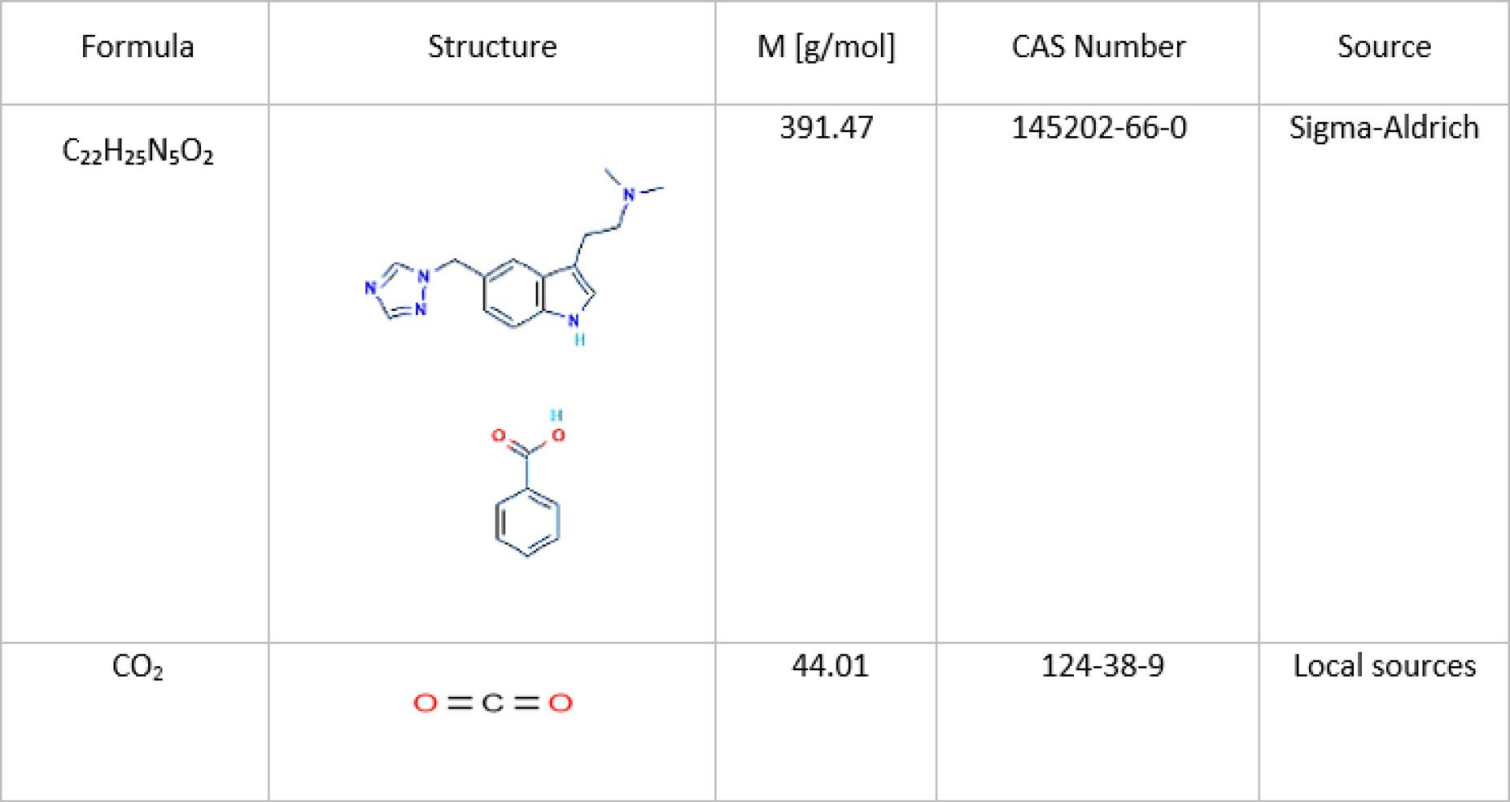
Properties of the materials.

### Solubility measurement procedure

A specialized high-pressure system is employed to measure the solubility of rizatriptan using gravimetric techniques in a supercritical CO_2_^6^. A 200 mL vessel capable of withstanding pressures up to 35 MPa and temperatures up to 423 K is prepared with leak-free seals, valves, and fittings. All valves and connectors were 1/8 inch in size. The system’s pressure and temperature sensors are calibrated for accuracy. Next, 2000 mg of rizatriptan powder is weighed precisely using an analytical balance with 0.01 mg of sensitivity. The powder is then compacted into 5-mm diameter tablets to ensure uniformity and structural integrity. The tablets are placed in a basket inside the vessel to maximize their exposure to SC-CO_2_. CO_2_ is introduced gradually using a high-precision pump (SC-1000, SUPEREX), with the pressure increased in 0.05 MPa increments to maintain stability. The pressure is monitored and held within ± 0.01 MPa. Temperature is controlled within ± 0.1 K using a thermocouple, and the mixture is stirred at 250 rpm to ensure uniformity. The setup runs for 240 min with continuous stirring to achieve solubility equilibrium, as determined by a preliminary test (Table S1). The pressure and temperature indicators are checked to ensure stability. After equilibration, the vessel is carefully depressurized to ambient conditions to halt dissolution without losing the sample. The remaining tablets are then removed and cleaned of residual CO_2_. If needed, they are dried in a desiccator. The undissolved drug is weighed using a high-precision balance. The experiment is replicated at least three times to calculate the mean solubility and standard deviation. The following formula was used to calculate the mass of dissolved rizatriptan:

1 $$\:{\text{m}}_{\text{d}\text{i}\text{s}\text{s}\text{o}\text{l}\text{v}\text{e}\text{d}}={\text{m}}_{\text{i}\text{n}\text{i}\text{t}\text{i}\text{a}\text{l}}-{\text{m}}_{\text{u}\text{n}\text{d}\text{i}\text{s}\text{s}\text{o}\text{l}\text{v}\text{e}\text{d}\:}\:$$

Include the molecular weights of rizatriptan (397.1 g/mol) and CO_2_ (44.01 g/mol) in the mole fraction calculations.

2 $$\:\text{M}\text{o}\text{l}\text{e}\:\text{o}\text{f}\:\text{d}\text{r}\text{u}\text{g}=\frac{{\text{m}}_{\text{d}\text{i}\text{s}\text{s}\text{o}\text{l}\text{v}\text{e}\text{d}}}{{\text{M}}_{\text{w},\:\:\:\text{r}\text{i}\text{z}\text{a}\text{t}\text{r}\text{i}\text{p}\text{t}\text{a}\text{n}\:\:}}$$

3 $$\:\text{M}\text{o}\text{l}\text{e}\:\text{o}\text{f}\:{\text{C}\text{O}}_{2}=\frac{{\text{m}}_{{\text{C}\text{O}}_{2}}}{{\text{M}}_{\text{w},\:{\:\text{C}\text{O}}_{2}\:\:}}$$

4 $$\:\text{y}=\frac{\text{M}\text{o}\text{l}\text{e}\:\text{o}\text{f}\:\text{r}\text{i}\text{z}\text{a}\text{t}\text{r}\text{i}\text{p}\text{t}\text{a}\text{n}\:\:}{(\text{M}\text{o}\text{l}\text{e}\:\text{o}\text{f}\:\text{r}\text{i}\text{z}\text{a}\text{t}\text{r}\text{i}\text{p}\text{t}\text{a}\text{n}\:\:+\text{M}\text{o}\text{l}\text{e}\:{\text{o}\text{f}\:\text{C}\text{O}}_{2})}$$

5 $$\:Solubility\:=\frac{\rho\:\times\:\:{M}_{rizatriptan}\:\times\:\:y}{{M}_{C{O}_{2}}\times\:\left(1-y\right)}$$

### Modeling of Rizatriptan solubility in SC-CO_2_

To provide a comprehensive framework for solubility prediction, this section elaborates on density-based and equation of state (EoS) models. It incorporates detailed explanations, parameter interpretations, and correlations from recent studies.

#### Density-based models

Density-based models are correlations that primarily relate solute solubility to the density of the SC-CO_2_, often incorporating temperature effects. These models have the advantage of simplicity, requiring fewer parameters than full thermodynamic models. They are particularly effective at correlating experimental data without requiring extensive physical property data, such as critical properties. They assume that solubility increases with solvent density due to enhanced solvation power near the critical point^33–36^. In this study, seven key models like Chrastil, Bartle et al., MST, based models such as Chrastil, Bartle, Kumar–Johnston (K–J), and Mendez–Santiago–Teja (MST), Bian et al. and Sodeifian et al. (Ⅰ and Ⅱ) and K–J are applied to rizatriptan solubility in SC-CO_2_ (Table 2).

**Table 2:** The list of models.

Equation	Density-based model
$\:\text{ln}{\text{y}}_{2}={a}_{0}+{a}_{1}ln{\rho\:}_{1}+\frac{{a}_{2}}{T}$	Chrastil^37^
$\:ln\left(\frac{{y}_{2}P}{{P}_{ref}}\right)={a}_{0}+\frac{{a}_{1}}{T}+{a}_{2}\left({\rho\:}_{1}-{\rho\:}_{ref}\right)$	Bartle et al.^19^
$\:Tln\left({y}_{2}P\right)={a}_{0}+{a}_{1}{\rho\:}_{1}+{a}_{2}T$	MST^38^
$\:ln{y}_{2}={a}_{0}+{a}_{1}{\rho\:}_{1}+\frac{{a}_{2}}{T}$	K-J^39^
${y_2}={\rho ^{({a_0}+{a_1}\rho )}}\exp (\frac{{{a_2}}}{T}+\frac{{{a_3}\rho }}{T}+{a_4})$	Bian et al.^40^
$\ln {\kern 1pt} {y_2}={a_0}+{a_1}\frac{{{P^{{\kern 1pt} 2}}}}{T}+{a_2}\ln (\rho T)+{a_3}(\rho \ln \rho )+{a_4}P\ln T+{a_5}\frac{{\ln \rho }}{T}$	Sodeifian et al. (Ⅰ)^41^
$\:\text{ln}{\text{y}}_{2}={a}_{0}+{a}_{1}\frac{{P}^{-0.5}}{T}+{a}_{2}\frac{ln{\rho\:}_{1}}{{T}^{2}}+{a}_{3}\frac{\text{l}\text{n}\left({\rho\:}^{-3}\right)}{T}$	Sodeifian et al. (Ⅱ)^42^

The Chrastil approach is grounded in chemical association principles, suggesting that a single solute molecule interacts with k solvent molecules to create a balanced solvato-complex. This leads to the logarithmic relationship, where the enthalpy term ($\:{a}_{2}$) combines solvation and vaporization effects

$$\:(\varDelta\:{H}_{t}=\varDelta\:{H}_{vap}+\:\varDelta\:{H}_{sol}).$$

The Bartle model extends the concept by normalizing solubility with pressure and using a reduced density term, allowing direct estimation of the solute’s heat of vaporization from parameter ($\:{a}_{2}$). It is particularly useful for systems exhibiting retrograde solubility. The MST model was derived from a Taylor expansion of the Helmholtz free energy near the solvent’s critical point, focusing on infinite dilution behavior. By incorporating the Clausius–Clapeyron equation for missing sublimation pressure data, it becomes a robust three-parameter tool. It excels in self-consistency checks, where plotting $\:T(\text{ln}\left(y.P\right)-{a}_{2}$) against density (ρ), should yield a single line for reliable data. The K–J model emphasizes the logarithmic dependence on density, reflecting clustering phenomena in supercritical fluids. Its parameters allow separation of entropic and enthalpic contributions, making it suitable for temperature extrapolation.

#### Equation of state modeling

EoS models provide a thermodynamic foundation for solubility predictions by equating fugacity between solid and supercritical phases, accounting for non-ideal behaviors at high pressures. For gas-solid equilibria, temperature (T), pressure (P), and fugacity ($\:{f}_{2}$) must be equal across phases:

6 $$\:{f}_{2}^{SC-C{O}_{2}}={f}_{2}^{solid}$$

The solute mole fraction ($\:{y}_{2}$) in SC-CO_2_ was estimated by the following equation:

7 $$\:{y}_{2}=\frac{{P}_{2}^{sub}\left(T\right)}{P}\frac{{\varnothing\:}_{2}^{sat}\left(T\right)}{{\varnothing\:}_{2}(T,P,y)}exp\left[\frac{{v}_{2}^{s}(P-{P}_{2}^{sub}\left(T\right))}{RT}\right]$$

Fugacity coefficients $\:{\varnothing\:}_{i}$ ​ are computed from the EoS via:

$$\:RTln\:{\varnothing\:}_{i}=-RTlnZ+{\int\:}_{V}^{\infty\:}\left[{\left(\frac{\partial\:P}{\partial\:{n}_{i}}\right)}_{T,V,{n}_{j}\ne\:{n}_{i}}-\frac{RT}{V}\right]dV$$

Models evaluated include PR, and SRK with binary parameters ($\:{l}_{ij}\:\text{a}\text{n}\text{d}\:{k}_{ij}$) fitted to data.

- Peng–Robinson (PR)

The PR EoS is a cubic model optimized for supercritical fluids, expressing pressure as:

8 $$\:P=\frac{RT}{v-b}-\frac{a\left(T\right)}{v\left(v+b\right)+b(v-b)}$$

9 $$\:a=0.45724\frac{{R}^{2}{T}_{c}^{2}}{{P}_{c}}\alpha\:({T}_{r},\omega\:)$$

10 $$\:b=0.0778\frac{R{T}_{c}}{{P}_{c}}$$

11 $$\:\alpha\:\left({T}_{r},\omega\:\right)={\left[1+k(1-\sqrt{{T}_{r})}\right]}^{2}$$

12 $$\:k=0.37464+1.5422\omega\:-0.26992{\omega\:}^{2}$$

PR excels in solubility modeling for drugs in SC-CO_2_, with $\:{k}_{ij\:}$often temperature-dependent for better fits.

b. Soave–Redlich–Kwong (SRK)

SRK, another cubic EoS, is defined as:

13 $$P = \frac{{RT}}{{\nu - b}} - \frac{{a(T)}}{{\nu \,\,(\nu + b)}}$$

Where,

14 $$a(T)=\frac{{0.42747{R^2}T_{c}^{2}}}{{{P_c}}}\, \times \,\underbrace {{{{(1+m(1 - T_{r}^{{0.5}}))}^2}}}_{{\alpha ({T_{r,\omega }})}}\,\,\,\,\,\,\,\,\,\,\,\& \,\,\,\,\,\,m=0.480+1.574\,\omega - 0.176\,{\omega ^2}$$

15 $$b = \frac{{0.08664RT_{c} }}{{P_{c} }}$$

The mixing rule of were defined as:^43^

16 $$\:{a}_{m}=\sum\:_{i}\sum\:_{j}{x}_{i}{x}_{j}{a}_{ij}$$

17 $$\:{b}_{m}=\sum\:_{i}\sum\:_{j}{x}_{i}{x}_{j}{b}_{ij}$$

Here, $\:{a}_{ij}$ and $\:{b}_{ij}$ were calculated as follows^43^:

18 $$\:{a}_{ij}={\left({a}_{i}{a}_{j}\right)}^{0.5}\left(1-{k}_{ij}\right)$$

19 $$\:{b}_{ij}=\left(\frac{{b}_{i}+{b}_{j}}{2}\right)\left(1-{l}_{ij}\right)$$

SRK is effective for CO_2_-drug solubilities, with modifications improving predictions for polar solutes.

#### Regular solution model

The regular solution model, which is predicated on solid-liquid equilibrium thermodynamics, was utilized to describe rizatriptan’s solubility in supercritical carbon dioxide. This model utilizes the fugacity ratio of the pure liquid solute to its solid form to calculate the solute’s activity in the liquid phase. This ratio is influenced by pressure and temperature. The activity coefficient is derived from the Flory–Huggins equation, which incorporates the solute’s molar volume, calculated via the Fedors method^44^ and the solubility parameter.

The regular solution model^45–50^ is employed to determine the following:

20 $$\ln \,\gamma _{{}}^{\infty }=\frac{{{\nu _{solute}}}}{{RT}}\,{({\delta _{scC{O_2}}} - \,{\delta _{solute}})^2}\,+\,1\, - \,\frac{{{\nu _{solute}}}}{{{\nu _{scC{O_2}}}}}\,+\,\ln \,(\frac{{{\nu _{solute}}}}{{{\nu _{scC{O_2}}}}})$$

The mole fraction is expressed as follows:

21 $$\ln {y_{solute}}=\frac{{\Delta H_{m}^{{}}}}{R}(\frac{1}{{{T_m}}} - \frac{1}{T}) - \,\,\frac{{{\nu _{solute}}}}{{RT}}\,\,{({\delta _{scC{O_2}}} - {\delta _{solute}})^2} - 1\,+\,(\frac{{{\nu _{solute}}}}{{{\nu _{scC{O_2}}}}}) - \ln \,(\frac{{{\nu _{solute}}}}{{{\nu _{scC{O_2}}}}})$$

An estimation of the solubility parameter ($\:{{\updelta\:}}_{{\:\text{C}\text{O}}_{2}}$) was conducted using the following equation:

22 $$\delta _{{scC{O_2}}}^{2}=\,{[\frac{{{\delta _{dref}}}}{{{{(\frac{{{\nu _{ref}}}}{{{\nu _{scC{O_2}}}}})}^{ - 1.25}}}}]^2}\,+\,\,{[\frac{{{\delta _{pref}}}}{{{{(\frac{{{\nu _{ref}}}}{{{\nu _{scC{O_2}}}}})}^{ - 0.5}}}}]^2}\,+\,{[\frac{{{\delta _{href}}}}{{\exp ( - 1.32 \times {{10}^{ - 3}}\,({T_{ref}} - T) - \ln \,{{(\frac{{{\nu _{ref}}}}{{{\nu _{scC{O_2}}}}})}^{0.5}})}}]^2}$$

It has been hypothesized that the presence of δ_solute_ may be indicative of a multifaceted role within the complex structures of various functions. The following are some of the proposed structural roles of the δs_olute_^20,51^:

23 $$\:{\delta\:}_{drug}=A+B\:{\rho\:}_{r,{CO}_{2}}$$

24 $$\:{\delta\:}_{drug}=A+B\:{\rho\:}_{r,{CO}_{2}}^{C}$$

25 $$\:{\delta\:}_{drug}=A+B\:{\rho\:}_{r,{CO}_{2}}+C\:{\rho\:}_{r,{CO}_{2}}^{2}$$

The solubility parameter of the solvent is estimated using specific equations, while δ_solute_ is modeled using temperature dependent expressions involving adjustable parameters (A, B, and C) and reference values. The model also requires the solute’s melting point (T_m_ = 450.1 K) and the enthalpy of fusion, which is estimated using group contribution methods^52^.

## Results and discussion

### Rizatriptan solubility in SC-CO_2_

The measurements were conducted at four isotherms (308.2 K, 318.2 K, 328.2 K, and 338.2 K) and five pressure points (12, 15, 18, 21, 24, 27, 30 MPa). Table 3 documents the experimental conditions, including temperature, pressure, SC-CO_2_ density, mole fraction, standard deviation, and solubility in grams per liter. Each solubility value is the mean of three measurements, with a consistent relative standard deviation of about 4.5%. These results demonstrate the high precision and reproducibility of the experimental methodology.

**Table 3:** Experimental mole fraction (y) of Rizatriptan in supercritical carbon dioxide.

T (K)	*P* (MPa)	ρ_CO2_ (kg/m^3^)	y × 10^4^	Standard deviation × 10^6^	Solubility (g/L)
308.0	12	768.52	0.090	0.370	0.0616
	15	816.10	0.121	0.506	0.0880
	18	816.10	0.141	0.524	0.1066
	21	874.55	0.177	0.518	0.1378
	24	895.66	0.227	0.599	0.1810
	27	913.70	0.258	0.727	0.2098
	30	929.85	0.286	0.869	0.2366
318.0	12	659.80	0.069	0.227	0.0406
	15	743.83	0.126	0.443	0.0835
	18	790.29	0.162	0.644	0.1140
	21	823.80	0.215	0.741	0.1577
	24	850.15	0.281	0.749	0.2127
	27	872.14	0.322	1.463	0.2500
	30	890.90	0.339	1.496	0.2687
328.0	12	506.75	0.045	0.231	0.0204
	15	654.94	0.099	0.561	0.0578
	18	724.13	0.176	0.743	0.1135
	21	768.74	0.241	0.736	0.1650
	24	801.92	0.302	1.055	0.2156
	27	828.51	0.353	1.349	0.2603
	30	850.83	0.380	1.010	0.2877
338.0	12	384.17	0.024	0.107	0.0083
	15	555.33	0.136	0.512	0.0675
	18	651.28	0.213	0.674	0.1236
	21	709.59	0.279	1.011	0.1763
	24	751.27	0.334	1.296	0.2233
	27	783.19	0.388	1.907	0.2705
	30	809.68	0.419	1.130	0.3018

**Fig. 1:**
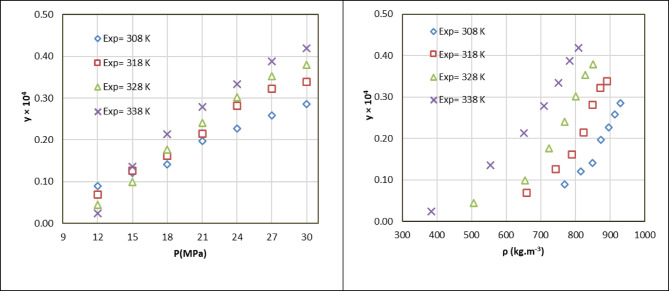
The experimental data verses pressure and density.

Table 3 presents the experimental data, showing mole fractions ranging from 0.240 × 10^−5^ to 4.19 × 10^−5^ and solubilities ranging from 0.0083 to 0.3018 g/L. Solubility increased monotonically with pressure at each isotherm due to enhanced SC-CO₂ density, which reduces intermolecular distances and strengthens solute-solvent interactions^33–36,53–55^. For instance, at 328.2 K, the mole fraction increased from 0.45 × 10^−5^ at 12 MPa (ρ = 506.8 kg/m^2^) to 3.80 × 10^−5^ at 30 MPa (ρ = 850.8 kg/m^2^), corresponding to a solubility rise from 0.0204 to 0.2877 g/L. Figure 1 presents a graphical representation of rizatriptan solubility as a function of pressure and SC-CO₂ density across the four isotherms. Each isotherm shows a steep increase in solubility with pressure, reflecting the enhanced solvating power of denser SC-CO_2_. The intersection of solubility curves between 12 and 15 MPa highlights the crossover region, where the effect of temperature shifts from reducing solubility (due to lower density) to enhancing it (due to higher vapor pressure). A secondary axis in Fig. 1 shows SC-CO_2_ density, emphasizing its role as a key determinant of solubility trends.

Temperature effects were complex due to the interplay of SC-CO_2_ density and rizatriptan’s sublimation vapor pressure. A crossover pressure of approximately 15 MPa was observed, at which point the solubility trends reversed. Below 15 MPa, solubility decreased with increasing temperature due to reduced solvent density (for example, at 12 MPa, ρ decreases from 768.5 kg/m^2^ at 308.2 K to 384.1 kg/m^2^ at 338.2 K). Above 15 MPa, solubility increased with temperature due to the dominant effect of higher sublimation vapor pressure (for example, at 30 MPa, y = 4.19 × 10^−5^ at 338.2 K vs. 2.04 × 10^−5^ at 308.2 K). This crossover phenomenon is characteristic of SCF systems and critical for process design. The maximum solubility (0.302 g/L) was observed at 338.2 K and 30 MPa, underscoring SC-CO_2_’s potential as a solvent for rizatriptan under these conditions. As the pressure rose at a constant temperature, the solubility of rizatriptan in supercritical carbon dioxide increased significantly. This behavior is primarily driven by the direct relationship between pressure and SC-CO_2_ density. As pressure increases, the density of SC-CO_2_ rises, which reduces the average intermolecular distance between CO_2_ molecules and increases the number of solvent molecules per unit volume. This enhanced density strengthens the interactions between rizatriptan and SC-CO_2_, thereby improving the solvent’s solvating capacity. For example, mole fraction significantly increased from 0.09 × 10^−4^ at 12 MPa to 0.286 × 10^−4^ at 30 MPa, at 308.2 K. Similar increases were observed across all tested isotherms, confirming the critical role of pressure in increasing the solubility. The influence of temperature on rizatriptan solubility is more complex due to the interplay of two competing factors. First, as temperature increases at constant pressure, the density of SC-CO₂ decreases, reducing its solvating power. Second, an increase in rizatriptan’s sublimation vapor pressure with rising temperature enhances solubility. This competition results in a crossover phenomenon, a hallmark of supercritical fluid systems^6^.

Below the crossover pressure: At pressures below 15 MPa, increasing temperature at a fixed pressure leads to decreased rizatriptan solubility. In this region, the reduction in SC-CO_2_ density dominates, diminishing the solvent’s ability to dissolve rizatriptan despite the increase in the solute’s vapor pressure. For example, at 12 MPa, the mole fraction decreased from 0.090 × 10^−4^ at 308.2 K to 0.024 × 10^−4^ at 338.2 K. Above the crossover pressure: At pressures above 15 MPa, an increase in temperature enhances solubility because the significant rise in rizatriptan’s sublimation vapor pressure outweighs the relatively minor decrease in SC-CO_2_ density. For example, at 30 MPa, the mole fraction increased from 0.286 × 10^−4^ at 308.2 K to 0.419 × 10^−4^ at 338.2 K.

### Solubility correlation results

In order to analyze and forecast rizatriptan’s solubility in SC-CO_2_, researchers employed seven established density-based models including Chrastil, Bartle et al., K-J, MST, Bian et al. and Sodeifian et al. (ⅠandⅡ) Models. These models establish a correlation between solubility and CO_2_ density as well as temperature, thereby circumventing the necessity of determining complex pure component properties or intricate binary interaction parameters. The performance of the model was evaluated by two statistical metrics such as AARD and R^2^ (Table 4). The AARD% is defined as the average absolute relative deviation, calculated as the mean percentage difference between experimental and predicted solubility values. R^2^ is the regression coefficient that quantifies how well the model explains the variability in the experimental data. The Chrastil Model is predicated on the premise of chemical association between solute and solvent molecules. The model is linearized to extract an association number and an apparent dissolution enthalpy. For rizatriptan, AARD = 11.88% and R^2^ = 0.967, indicating a strong agreement with experimental data. The Bartle et al. Model^13^ was constructed on the Chrastil framework, but an additional term was incorporated to account for the temperature dependence of the association number. The model demonstrated a comparable performance to the Chrastil model, with an AARD of approximately 10.58% and a R^2^ of approximately 0.975. The K–J Model is applicable across diverse fields, and its flexible functional form suits complex systems. The model demonstrated the optimal fit for rizatriptan solubility, with AARD = 8.04% and R^2^ = 0.989, thereby reflecting a high degree of precision in describing the solubility-SC-CO_2_ relationship. The MST model explains that solubility thermodynamics is contingent upon the presence of strong solute-solvent interactions within the system. For rizatriptan, the AARD is approximately 12.01%, and the R^2^ is approximately 0.977. This indicates a solid but slightly less precise fit than the Sodeifian et al. and K–J model.

In addition, the self-consistency test has been assessed by MST model. It shows a nearly linear relationship between temperature-dependent solubility parameters. However, deviations occur at densities below approximately half the critical density of CO_2_, where solvent power decreases. This linearity allows for reliable solubility extrapolation and serves as a tool for evaluating the consistency of experimental data. When solubility data are consistent, plotting T[ln(y×P) – a_2_] versus density (ρ) yields a straight line. Several researchers have used this approach to verify the reliability of data in supercritical CO₂ systems. For example, Shojaee et al.^56^ confirmed the linear behavior for carvedilol, Tamura et al.^49^ observed similar consistency for 1-aminoanthraquinone and 1-nitroanthraquinone, and Ahmadi Sabegh et al.^57^ validated the solubility of amoxicillin using the same graphical method. Overall, the model provides a practical means of validating data and predicting solubility trends in supercritical fluids.

**Fig. 2:**
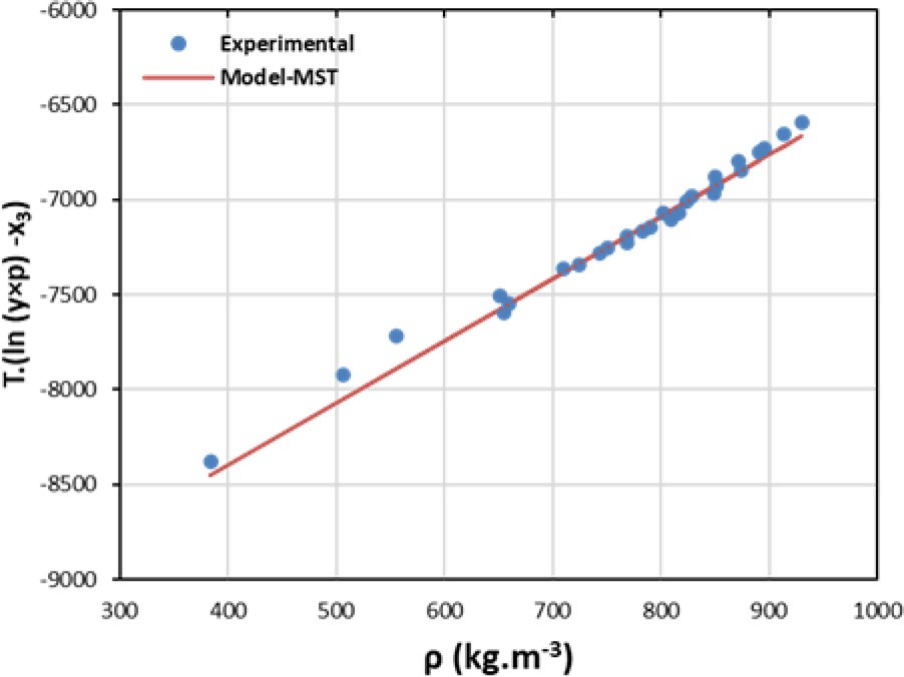
Self-consistency test by MST models results.

**Fig. 3:**
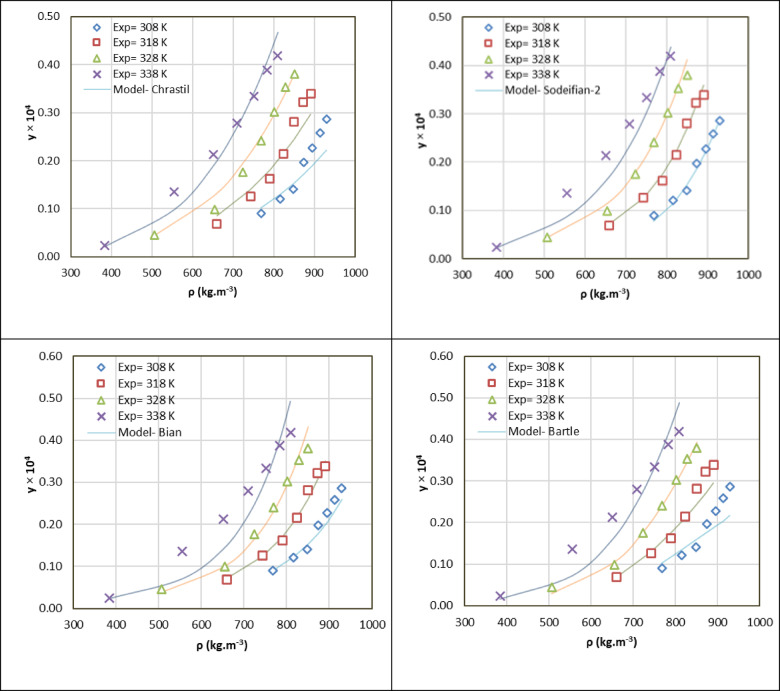

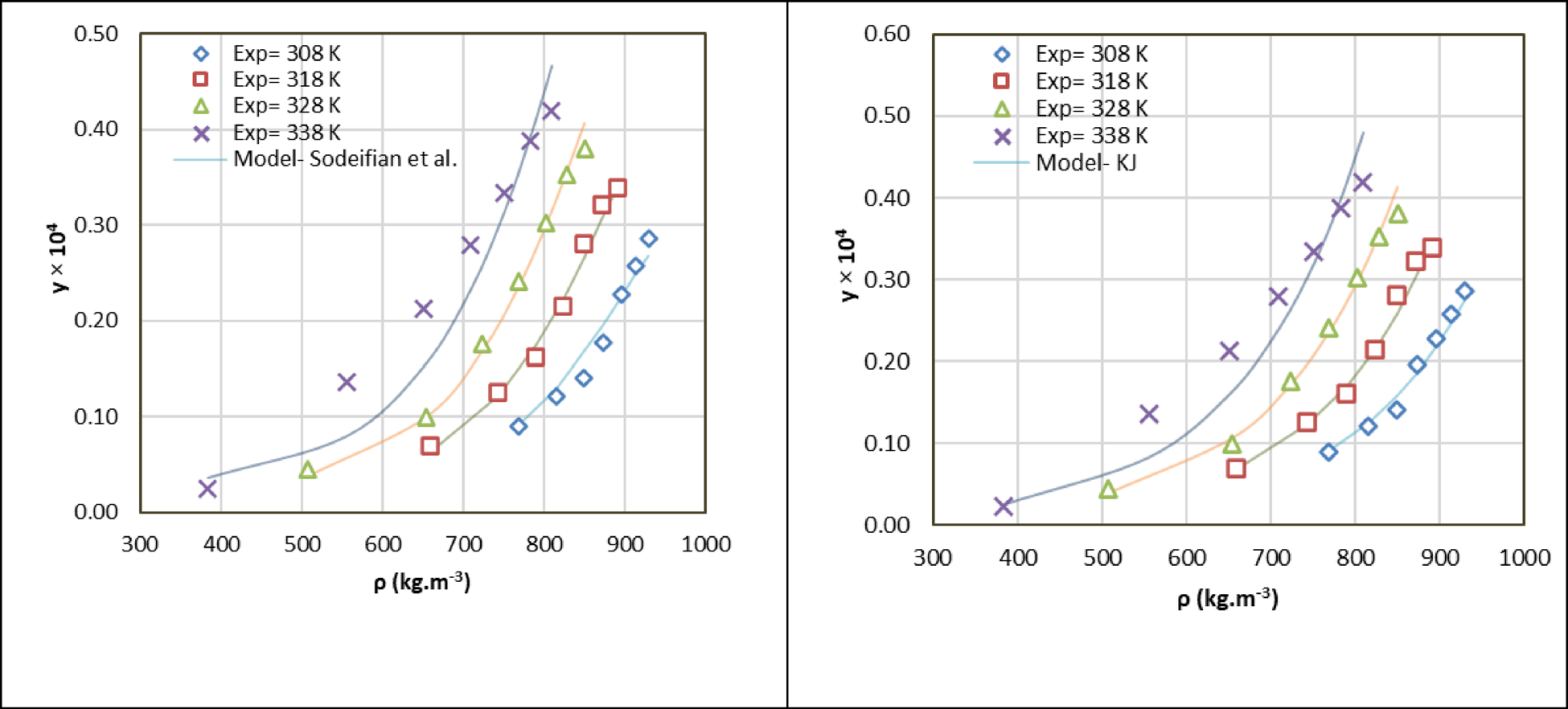
density based models results.

Table 4 provides a comprehensive overview of the performance metrics and fitted parameters for all models. The Sodeifian et al.Ⅱ model with an AARD of 7.78% and a R^2^ of approximately 0.990 is distinguished by its superior performance metrics a finding that is further accentuated in Fig. 3, which demonstrates a close correspondence between the experimental and predicted solubility values.

**Table 4:** The parameters of the desired empirical models.

Model	Adjustable parameters						Criteria parameters	
	a	B	c	d	e	f	AARD (%)	*R* ^2^
Chrastil	5.16	− 22.12	− 4577.4	–	–	–	11.88	0.979
Bartle	15.81	0.011	− 7162.7	–	–	–	13.78	0.943
KJ	− 1.24	− 4852.7	0.0069	–	–	–	8.04	0.989
MST	− 9760.2	3.27	16.78	–	–	–	12.01	0.977
Bian et al.	− 0.0489	0.0035	259.5	6.53	− 14.24		10.39	0.981
Sodeifian et al. Ⅰ	18.459	− 0.00618	− 1.46	0.002	− 0.0054	− 1029.4	12.29	0.967
Sodeifian et al. Ⅱ	− 28.97	− 2182.1	− 4268	− 759.3	–	–	7.78	0.990

Modeling rizatriptan’s solubility in SC-CO_2_ using the EoS and modified regular solution model requires a comprehensive set of critical physicochemical properties for the rizatriptan and the SC-CO_2_ (for example, critical temperature (T_c_= 1119.4 K), critical pressure (P_c_= 24.56 bar), acentric factor (ω = 0.894), sublimation pressures, and physicochemical properties such as melting enthalpy (29.73 kJ/mol) and molar volume (273.7 cm^2^/mol). These properties were estimated using methods such as the Mario-Gani^58^, Ambrose-Walton^59^, and Fedors^60^ approaches. These properties are typically derived through well-established group contribution methods. These methods estimate molecular properties by analyzing the contributions of functional groups within the molecule, providing a reliable foundation for thermodynamic modeling. These properties, along with other relevant rizatriptan parameters, are systematically documented in Table 5 and serve as a reference for the modeling efforts in this study.

**Table 5:** Properties of Rizatriptan.

T_b_ (K)^a^	T_c_ (K)^a^	*P*_c_ (bar)^a^	ω^b^	$\Delta H_{m}^{{}}$ (kJ. kmol^− 1^) ^a^	v_s_ (cm^3^.mol^− 1^)^c^	*P*^sub^ (Pa)^b^; T = 308–338 (K)			
851.41	1119.4	24.56	0.894	29.73	273.7	2.47e^− 10^	1.88e^− 9^	1.22e^− 8^	6.60e^− 8^

The solubility correlation results for rizatriptan in SC-CO_2_ are detailed in Table 6. This table presents key performance metrics for two widely used EoS models: the PR and SRK equations of state. Both models employ the van der Waals 2 (vdW2) mixing rule. The table includes optimized binary interaction parameters (k_12_ and l_12_), which account for intermolecular interactions between rizatriptan and SC-CO_2_; the AARD%, which quantifies the deviation between experimental and predicted solubility values; and the R^2^, which indicates how well the model fits the experimental data. Specifically, Table 6 reports R^2^ values for the PR and SRK models at four temperatures (308.15 K, 318.15 K, 328.15 K, and 338.15 K). The SRK-vdW2 model consistently has higher R^2^ values across all temperatures, demonstrating its superior ability to correlate rizatriptan solubility in SC-CO_2_ compared to the PR-vdW2 model. The SRK model’s R^2^ values approach unity, indicating a closer alignment with experimental data and reflecting its enhanced predictive accuracy over the entire temperature range studied.

Table 6 serves as a centralized summary of the solubility correlation outcomes for the PR-vdW2 and SRK-vdW2 models. It includes the optimized binary parameters (k_12_ and l_12_), which are fine tuned to minimize discrepancies between experimental and modeled solubility values. The SRK-vdW2 model’s consistently lower AARD% and higher R^2^ values underscore its robustness and reliability in capturing the solubility behavior of rizatriptan in SC-CO_2_.

Both the PR and SRK models are rooted in a rigorous thermodynamic framework, leveraging the strengths of equations of state to describe complex phase behavior in supercritical systems. By relying on the thermodynamic principles of EoS models, both PR and SRK provide a physically grounded approach that avoids the limitations of empirical correlations, such as poor extrapolation beyond the experimental data range. This makes them particularly suitable for applications requiring predictions under varying conditions, such as process optimization in supercritical fluid technologies.

**Table 6:** Outcome of modeling of Rizatriptan solubility by PR.

Model	Parameter	T = 308 K	T = 318 K	T = 328 K	T = 338 K	Mean
PR-vdW2	*k*_12_	− 0.0302	− 0.0762	− 0.1665	− 0.229	
	*l*_12_	− 0.240	− 0.346	− 0.568	− 0.638	
	AARD %	11.86	16.51	20.96	21.69	17.75
	R^2^	0.979	0.968	0.961	0.956	0.966
SRK-vdW2	*k*_12_	− 0.014	− 0.031	− 0.118	− 0.221	
	*l*_12_	− 0.179	− 0.286	− 0.506	− 0.815	
	AARD %	12.59	17.18	21.87	24.91	19.14
	R^2^	0.969	0.954	0.949	0.946	0.954

**Fig. 4:**
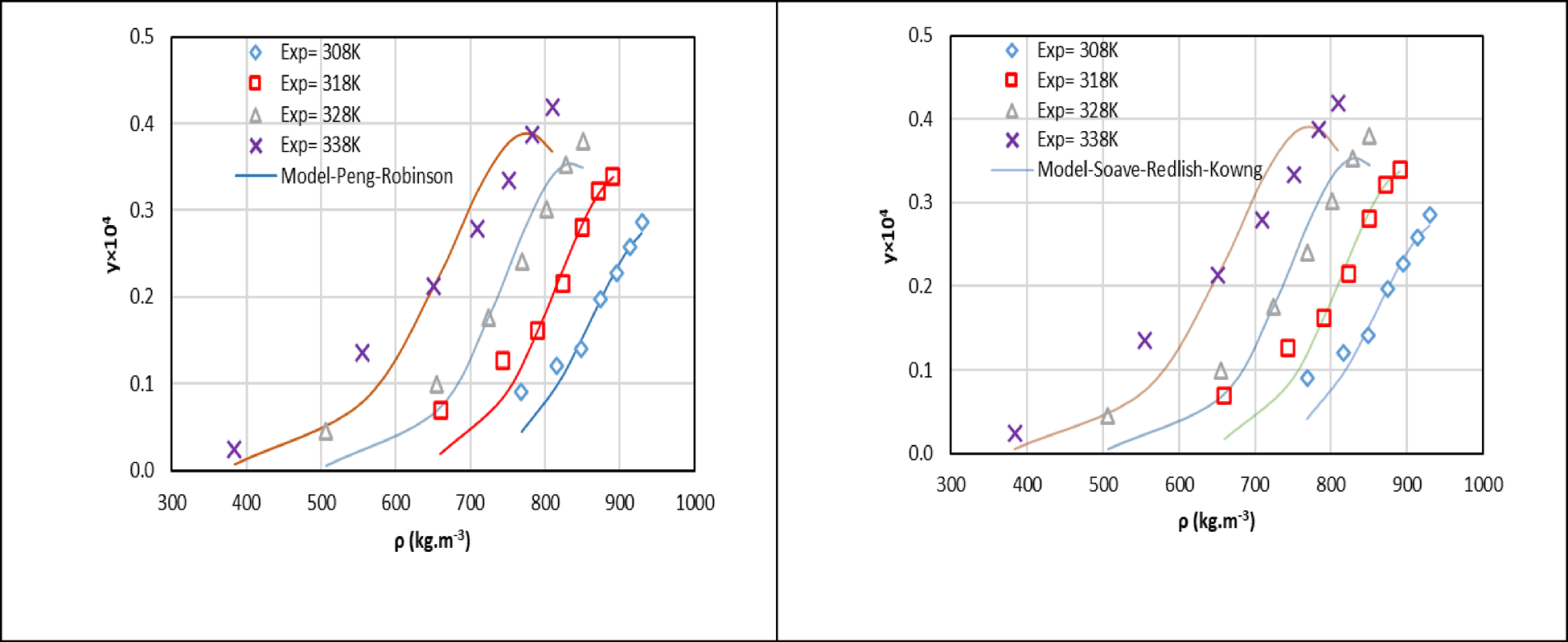
PR and SRK models outcome for mole fraction of rizatriptan.

Figures 4 and S1 illustrate the comparison between experimental rizatriptan solubility data and model predictions, which further validates the superior performance of the PR-vdW2 model. Figure 4 shows that the PR-vdW2 model aligns more closely with experimental solubility values than the SRK-vdW2 model.

This study thoroughly assesses the thermodynamic consistency of the PR equation of state by validating the fitted parameters for rizatriptan solubility in supercritical CO₂ using the van der Waals 2 (vdW2) mixing rule. Key results show that the binary interaction parameters *k*_12_ and *l*_12_ become more negative with increasing temperature (e.g., decreasing from − 0.0302 to − 0.240 at 308 K to − 0.229 and − 0.638 at 338 K). This suggests a weakening of solute-solvent interactions as density and cohesive energy decrease. This is an expected thermodynamic trend. The regression shows smooth parameter changes, supporting numerical stability. Model performance metrics reveal strong agreement with experimental solubility data. The PR-vdW2 model slightly outperforms the SRK-vdW2 model with lower AARD (17.75%) and higher mean R^2^ (0.966). The PR-vdW2 model also maintains high-pressure consistency, displaying the expected crossover behavior with temperature: solubility increases with pressure at each isotherm. This captures the effects of temperature on solvent power and solute vapor pressure, as well as CO_2_ density increases. Overall, the PR-vdW2 model is numerically stable and thermodynamically coherent for rizatriptan solubility in SC-CO_2_. Furthermore, applying the Valderrama et al.^61^ method to four isotherms (308.2–338.2 K; 28 points) yields TC classifications of % Δy_2_ <20% and |%ΔA (avg)| of 7.8–8.7%. All isotherms pass the Gibbs–Duhem area consistency test. These results support the manuscript’s conclusion that the PR-vdW2 model reliably and consistently models rizatriptan solubility in supercritical carbon dioxide.

To correlate the experimentally determined solubility of rizatriptan in SC-CO_2_, a modified regular solution models were employed. The performance of the model was evaluated using statistical metrics to ensure its accuracy and reliability (AARD and R^2^). The results of these analyses are presented in Table 7. The modified regular solution models showed excellent agreement with the experimental solubility data. The solubility parameters have been calculated in Table S2. Specifically, the models achieved an AARD of approximately 6.89%, indicating a low level of error in predicting solubility values. Additionally, the average R^2^ of 0.991 reflects a high degree of correlation between the experimental and predicted data. These statistical outcomes highlight the model’s ability to capture the rizatriptan-CO_2_ system’s underlying thermodynamic behavior.

**Table 7:** The parameters of the regular solution method.

Various δ_solute_ definitions	Adjustable parameters			AARD%	*R* ^2^
	A	B	C		
${\delta _{solute}}=\,A\,+\,B\rho _{{r,solvent}}^{{}}$	-12.20	7.57	-	09.10	0.980
${\delta _{solute}}=\,A\,+\,B\rho _{{r,solvent}}^{c}$	-10.54	6.04	1.16	8.76	0.987
${\delta _{solute}}=\,A\,+\,B\rho _{{r,solvent}}^{{}}\,+\,C\rho _{{r,solvent}}^{2}$	-11.09	6.147	0.457	6.89	0.991

**Fig. 5:**
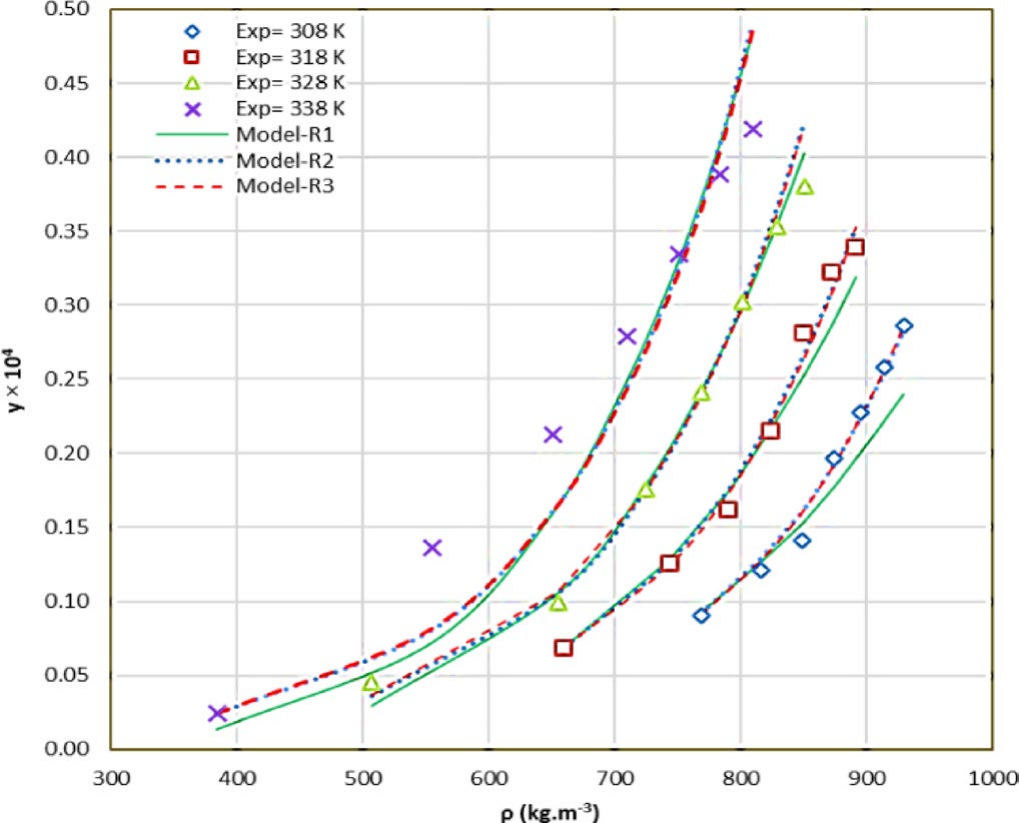
Correlating solubility of rizatriptan using the regular solution.

## Conclusion

This study provides a comprehensive analysis of rizatriptan solubility in SC-CO_2_ to advance sustainable pharmaceutical processing. Experimental solubility measurements conducted at temperatures ranging from 308.2 K to 338.2 K and pressures from 12 to 30 MPa revealed mole fractions ranging from 0.24 × 10^−5^ to 4.19 × 10^−5^ and dissolution concentrations from 0.008 g/L to 0.302 g/L, with a crossover pressure identified at approximately 15 MPa. These results highlight the significant influence of SC-CO_2_ density and rizatriptan’s sublimation vapor pressure on solubility behavior, with pressure driven density increases enhancing solute-solvent interactions and temperature effects exhibiting a complex interplay due to competing density and vapor pressure dynamics. seven density-based models (Chrastil, Bartle, Kumar–Johnston, Mendez–Santiago–Teja, Bian et al., and Sodeifian et al. (Ⅰand Ⅱ)) and three thermodynamic models (Peng–Robinson, Soave–Redlich–Kwong, and regular solution) were employed to correlate the experimental data. The Kumar–Johnston model exhibited the highest accuracy among the density-based models (AARD = 8.04%, R^2^ = 0.989), while the regular solution model outperformed the equations of state (AARD = 6.89%, R^2^ = 0.991), demonstrating robust predictive capabilities. Thermodynamic analysis further elucidated key properties, including an overall enthalpy of 38.06 kJ/mol, vaporization enthalpy of 50.55 kJ/mol, and solvation enthalpy of − 12.49 kJ/mol, providing critical insights into rizatriptan’s phase behavior in SC-CO_2_. These findings establish a reliable foundation for optimizing supercritical fluid technologies, such as antisolvent precipitation and particle engineering, to enhance rizatriptan’s solubility and bioavailability while promoting environmentally friendly pharmaceutical manufacturing. The validated experimental data and predictive models offer valuable tools for the rational design and scaling of sustainable processes, including micronization, coprecipitation, and encapsulation, ultimately contributing to improved drug delivery systems and therapeutic efficacy for migraine treatment.

## Supplementary Information

Below is the link to the electronic supplementary material.

Supplementary Material 1

## Data Availability

The datasets used and/or analysed during the current study available from the corresponding author on reasonable request.
