# Supplementary material for: Measurement and modeling of rizatriptan in supercritical CO2 for pharmaceutical processing
Source: Sci Rep. 2025 Nov 26;15:42267. doi: 10.1038/s41598-025-26435-w (PMC12657942; doi:10.1038/s41598-025-26435-w)
Supplement: Supplementary file 1 — Supplementary Material 1 [file 41598_2025_26435_MOESM1_ESM.docx]

**Measurement and Modeling of Rizatriptan in Supercritical CO₂ for Pharmaceutical Processing**

Sami Bawazeer*

Department of Pharmaceutical Sciences, Faculty of Pharmacy, Umm Al-Qura, University, Makkah, Saudi Arabia

Email: [sbawazer@proton.me](mailto:sbawazer@proton.me)

Table S1. Preliminary tests.

| T | P | Time | Solubility (g/l) |
| --- | --- | --- | --- |
| 338 | 30 | 60 | 0.0788±0.0007 |
| 338 | 30 | 90 | 0.1676±0.0008 |
| 338 | 30 | 120 | 0.2129±0.0008 |
| 338 | 30 | 150 | 0.2790±0.0011 |
| 338 | 30 | 180 | 0.2858±0.0012 |
| 338 | 30 | 210 | 0.3018±0.0014 |
| 338 | 30 | 240 | 0.3018±0.0015 |

Table S2. Solubility parameters with different equation.

| T(K) | P(MPa) | **ρ_CO2_ (kg/m^3^)** | δ (Eq. 23) | δ (Eq. 24) | δ (Eq. 25) |
| --- | --- | --- | --- | --- | --- |
| 308.0 | 12 | 768.52 | 11.6 | 11.6 | 11.6 |
| 308.0 | 15 | 816.10 | 12.4 | 12.4 | 12.4 |
| 308.0 | 18 | 816.10 | 12.9 | 12.9 | 12.9 |
| 308.0 | 21 | 874.55 | 13.3 | 13.3 | 13.3 |
| 308.0 | 24 | 895.66 | 13.6 | 13.6 | 13.6 |
| 308.0 | 27 | 913.70 | 13.9 | 13.9 | 13.9 |
| 308.0 | 30 | 929.85 | 14.2 | 14.2 | 14.2 |
| 318.0 | 12 | 659.80 | 9.9 | 9.9 | 9.9 |
| 318.0 | 15 | 743.83 | 11.2 | 11.2 | 11.2 |
| 318.0 | 18 | 790.29 | 11.9 | 11.9 | 11.9 |
| 318.0 | 21 | 823.80 | 12.5 | 12.5 | 12.5 |
| 318.0 | 24 | 850.15 | 12.9 | 12.9 | 12.9 |
| 318.0 | 27 | 872.14 | 13.2 | 13.2 | 13.2 |
| 318.0 | 30 | 890.90 | 13.5 | 13.5 | 13.5 |
| 328.0 | 12 | 506.75 | 7.7 | 7.7 | 7.7 |
| 328.0 | 15 | 654.94 | 9.8 | 9.8 | 9.8 |
| 328.0 | 18 | 724.13 | 10.9 | 10.9 | 10.9 |
| 328.0 | 21 | 768.74 | 11.6 | 11.6 | 11.6 |
| 328.0 | 24 | 801.92 | 12.1 | 12.1 | 12.1 |
| 328.0 | 27 | 828.51 | 12.5 | 12.5 | 12.5 |
| 328.0 | 30 | 850.83 | 12.9 | 12.9 | 12.9 |
| 338.0 | 12 | 384.17 | 6.0 | 6.0 | 6.0 |
| 338.0 | 15 | 555.33 | 8.4 | 8.4 | 8.4 |
| 338.0 | 18 | 651.28 | 9.8 | 9.8 | 9.8 |
| 338.0 | 21 | 709.59 | 10.6 | 10.6 | 10.6 |
| 338.0 | 24 | 751.27 | 11.3 | 11.3 | 11.3 |
| 338.0 | 27 | 783.19 | 11.8 | 11.8 | 11.8 |
| 338.0 | 30 | 809.68 | 12.2 | 12.2 | 12.2 |


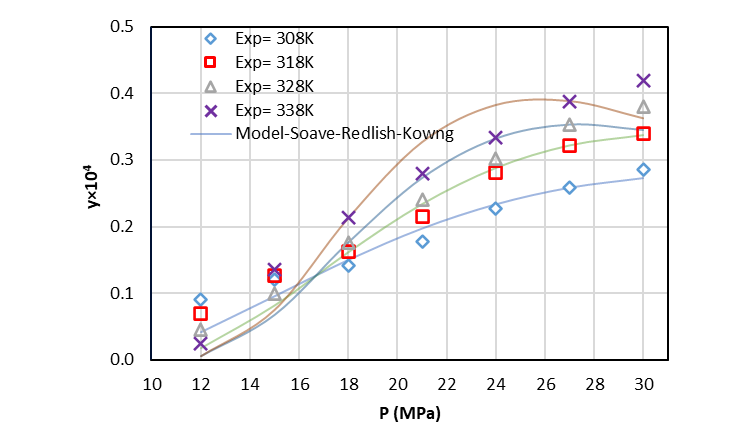

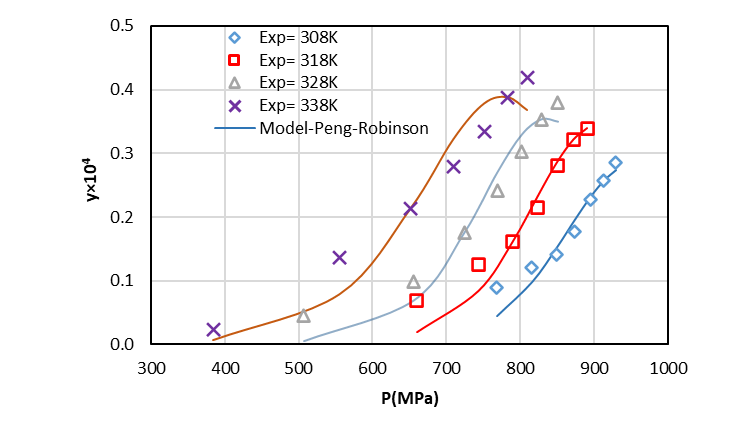


Figure S1. PR and SRK models outcome for mole fraction of rizatriptan vs pressure.
